# A Cross-Talk between the Erythrocyte L-Arginine/ADMA/Nitric Oxide Metabolic Pathway and the Endothelial Function in Subjects with Type 2 Diabetes Mellitus

**DOI:** 10.3390/nu13072306

**Published:** 2021-07-04

**Authors:** Damian Gajecki, Jakub Gawryś, Jerzy Wiśniewski, Paulina Fortuna, Ewa Szahidewicz-Krupska, Adrian Doroszko

**Affiliations:** 1 Department of Internal Medicine, Hypertension and Clinical Oncology, Wroclaw Medical University, 50-556 Wroclaw, Poland; damian.gajecki@umed.wroc.pl (D.G.); jakub.gawrys@umed.wroc.pl (J.G.); ewa.szahidewicz-krupska@umed.wroc.pl (E.S.-K.); 2 Central Laboratory of Instrumental Analysis, Wrocław University of Science and Technology, Wybrzeże Wyspiańskiego 27, 50-370 Wrocław, Poland; jerzy.wisniewski@pwr.edu.pl; 3 Department of Medical Biochemistry, Wroclaw Medical University, 50-368 Wroclaw, Poland; paulina.fortuna@umed.wroc.pl

**Keywords:** erythrocyte, nitric oxide, ADMA, type 2 diabetes mellitus, endothelial dysfunction

## Abstract

(1) Background: Type-2-diabetes-mellitus (DM) is one the most important cardiovascular-risk-factors. Among many molecules regulating vascular tone, nitric oxide appears to be the most pivotal. Although micro- and macrovascular-abnormalities are extensively studied, the alterations in the nitric-oxide-metabolic-pathway require further investigations. Additionally, the role of erythrocytes in the vascular tone regulation has not been extensively explored. The aim of this study was to evaluate the endothelial-function and the nitric-oxide-metabolic-pathway in erythrocytes and plasma of diabetic individuals. (2) Methods: A total of 80 subjects were enrolled in this cross-sectional study, including 35 patients with DM and 45 healthy individuals. The endothelial-function was evaluated in response to different stimuli. (3) Results: In the DM group, decreased Arginine and citrulline concentrations in the plasma compartment with reduced Arginine/ADMA and ADMA/DMA-ratios were observed. Preserved nitric-oxide-metabolism in erythrocytes with reduced citrulline level and significantly higher NO-bioavailability were noted. Significant endothelial dysfunction in DM individuals was proved in response to the heat-stimulus. (4) Conclusions: DM patients at an early stage of disease show significant differences in the nitric-oxide-metabolic-pathway, which are more pronounced in the plasma compartment. Erythrocytes constitute a buffer with a higher nitric-oxide-bioavailability, less affected by the DM-related deviations. Patients at an early-stage of DM reveal endothelial-dysfunction, which could be diagnosed earlier using the laser-Doppler-flowmetry.

## 1. Introduction

Type 2 diabetes mellitus (DM) is one of the most important health emergencies in the 21st century. The population suffering from DM is estimated for 463 million worldwide and is rapidly growing. Predictions say that the number may reach 578 million by 2030, and 700 million by 2045 [1].

DM is associated with higher relative risk of cardiovascular disorders (CVD) which is estimated between 1.6 and 2.6 and slightly higher in women [2]. Cardiovascular disease in DM patients occurs approximately 15 years earlier than in healthy subjects. It is the main cause of mortality in this group which is different based on sex-dependence. CVD mortality in men with diabetes increases 1–3 times compared to diabetes-free individuals, while the same coefficient estimated in women varies from 2 to 5 times [3]. It is not clear what plays the major role in inducing the differences, but the most important factor seems to be the attenuation of estrogens protective influence against cardiovascular complications in DM women. It was proved that DM impairs endothelial response in women to a greater extent than among males [4]. DM diminishes antiproliferative effects of estrogen on the vascular smooth muscle cells by modifying expression, activity, and balance between the estrogen receptors (ER): ERα and ERβ. Domination of ERβ over ERα activity results in increased inflammatory profile, excessive reactive oxygen species (ROS) formation, and aggravated atherosclerotic plaque formation [5]. In that way, estrogens increase the nitric oxide (NO) bioavailability reducing NO inactivation and additionally may directly increase NO generation [6]. Furthermore, diabetic females are characterized by worse cardiovascular profile, including higher average HbA1c, LDL cholesterol, and BMI levels [4]. Catalan et al. proved that, despite the fact that men more often present carotid plaques in general, in a subgroup of newly diagnosed diabetic women, carotid atherosclerosis was more prevalent [7].

Cardiovascular risk in DM patients is highly dependent on endothelial dysfunction as well as micro- and macrovascular complications coexisting in DM. Among numerous factors, nitric oxide (NO) bioavailability and its metabolic pathway abnormalities seem to be the most crucial. Asymmetric dimethylarginine (ADMA) is a pivotal element leading to the NO synthesis disturbances by competitive inhibiting the nitric oxide synthase (NOS). Significant evidence indicates that not only ADMA concentration per se decides on endothelial function but also the decreased Arginine level and the Arginine/ADMA ratio seems to be additional and potentially more sensitive markers of endothelial dysfunction. The Arginine/ADMA ratio as an indicator of reduced global bioavailability of Arginine and NO production was postulated to be an independent CVD risk factor and to correspond with the severity of hypertension, congestive heart failure, and atherosclerosis [8,9].

Up to date, most of the studies have focused on the NO metabolism abnormalities being seen in the plasma compartment of diabetic subjects. Hardly any of them aims to evaluate the erythrocytes’ role in this process. As the red blood cells (RBC) remain in constant contact with the endothelium and enable nitric oxide transport into distant hypoxic areas, their role in regulating the nitric oxide bioavailability might be underestimated. For many years erythrocytes have been considered to just eliminate the NO, which easily reacts with hemoglobin. However, recent studies pointed out that erythrocytes contain NOS and are able to produce NO. Subsequently they release it into the vasculature, regulating the blood flow in distal hypoxic regions [10]. It demonstrates that the conventional theory needs to be verified and the specific role of erythrocytes in the nitric oxide metabolism and its influence on endothelial dysfunction requires detailed evaluation. 

Hence, the main goal of this study was to evaluate the nitric oxide metabolism abnormalities in plasma and erythrocytes of diabetic subjects with a close assessment of endothelial function using different tools. Additionally, it was intended to define which of the nitric oxide metabolites reflects better endothelial dysfunction at the early stages of diabetes mellitus. 

## 2. Materials and Methods

### 2.1. Ethical Approval

All experiments were conducted and approved in accordance with the guidelines of the Bioethics Committee at the Wroclaw Medical University (KB-155/2019) from 28 February 2019 and adhered to the principles of the Declaration of Helsinki (Seventh Revision, 64th World Medical Association meeting, Fortaleza, 2013). All of the individuals agreed to participate in the study by signing a written informed consent. 

### 2.2. Patients

A total of 100 patients were investigated in the study. The inclusion criteria comprised diabetes mellitus diagnosed according to the American and Polish Diabetes Associations criteria, treated with oral metformin at age of 35–80 years. Subjects with the presence of diabetic complications, including microangiopathy, macrovascular diseases, past history of stroke, or myocardial infarction, as well as taking anticoagulant or antiplatelet treatment, were excluded from this study. In order to exclude the potential variables affecting the differences between groups, we excluded subjects with concomitant hypertension. Finally, a total of 80 consecutive subjects met properly the inclusion and exclusion criteria and were enrolled to the study, including 35 patients with diabetes mellitus (female: 12, male: 23) and 45 healthy individuals qualified to the control group, respectively.

**Scheme 1:**
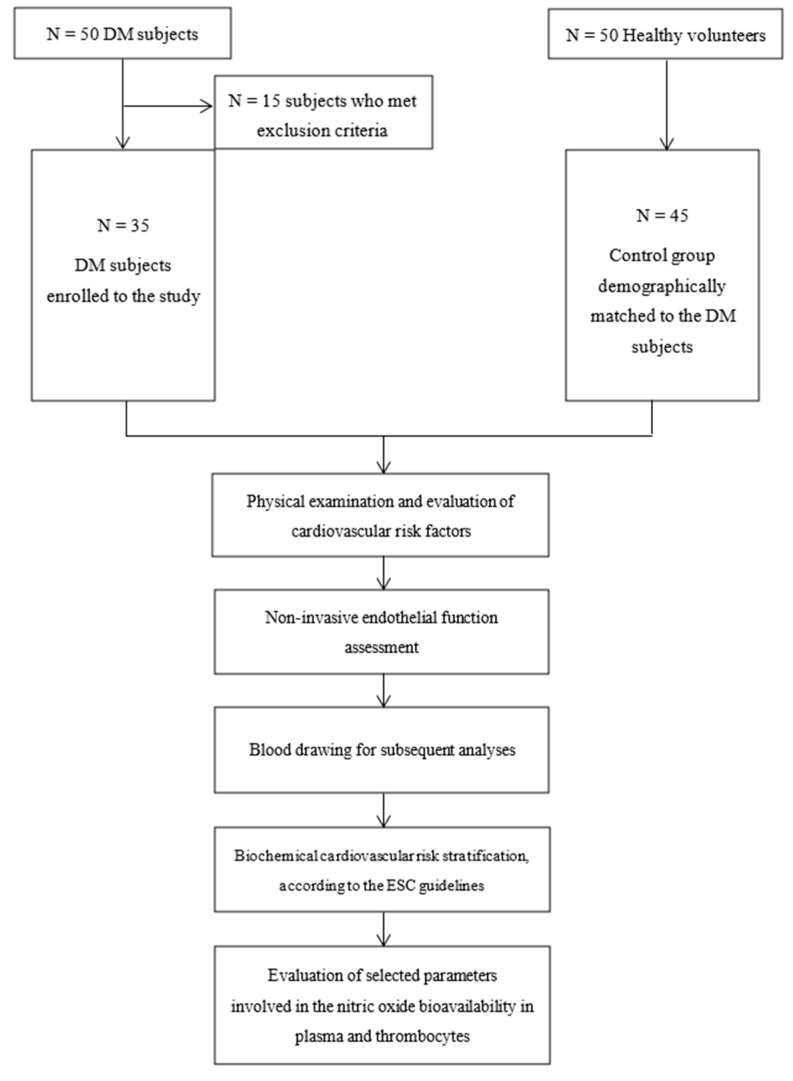
A flow chart presenting the study protocol.

The control group was recruited from outpatient clinics in pursuance of demographic characteristics (age, sex, region), meeting the inclusion and exclusion criteria properly. All of them had previously undergone the screening for the presence of the glucose metabolism alterations, including diabetes, impaired fasting glycemia, impaired glucose tolerance, and insulin resistance. Subjects with any of these disturbances were excluded from the control group. All of the participants underwent a standard detailed physical examination (Scheme 1).

### 2.3. Blood Sample Collection

Forty-four milliliters of peripheral blood was collected using the Sarstedt S-Monovette^®^ system (Sarstedt AG & Co., Nümbrecht, Germany). The sample (1.6 mg EDTA/mL blood) tubes within 30 min after the collection, were centrifuged at 1000× *g* for 15 min at 4 °C and stored at −80 °C until further analysis. 

### 2.4. Erythrocytes Preparation

The erythrocytes were removed from the plasma within the 10 min following blood drowning. Erythrocytes samples were thawed on ice. Subsequently, a 10 µL of internal standards solution and a 1200 µL of cold extraction solution containing methanol, acetonitrile, and water (5:3:2) were added and vortexed (for 15 min, 1200 rpm at 4 °C). Samples were centrifuged (for 15 min, 22,000 rpm at 4 °C) and the supernatants were transferred into new microtubes. Samples were then dried at 55 °C and afterwards dissolved in 100 µL of water and vortexed (for 5 min, 1200 rpm at 25 °C). Subsequently, 50 µL of borate buffer (0.025 M Na_2_B_4_0_7_ × 10H_2_O, 1.77 mM NaOH, pH = 9.2), 400 µL of acetonitrile, and 10 µL of 10% benzoyl chloride (BCl) in acetonitrile were added and vortexed again (for 10 min, 1200 rpm, at 25 °C). After derivatization samples were dried at 55 °C using the SpeedVac evaporator. Dried samples were reconstituted in 50 µL of 3% of methanol in water and centrifuged (for 10 min, 15,000 rpm, at 4 °C). Clear supernatant was transferred into chromatographic polypropylene vial with attached 200 µL insert.

### 2.5. Plasma Preparation

Plasma concentrations of metabolites involved in nitric oxide (NO) synthesis were measured according to method described by Fleszar et al. [11]. Briefly, a 100 μL of plasma, 50 µL of borate buffer, 10 µL of internal standard solution (100 µM D7-Arginine, 20 µM D7-ADMA, 25 µM D6-DMA, 100 µM D6-ornithine, and 50 µM D4-citrulline) were transferred into the 2 mL polypropylene tubes and mixed (for 1 min, 1200 rpm, at 25 °C). Then, 400 µL of acetonitrile and 10 µL of 10% BCl in acetonitrile were added and mixed (for 10 min, 1200 rpm, at 25 °C). Subsequently, samples were centrifuged (for 7 min, at 4 °C, 22,000 RCF) and 100 µL of clear supernatant was diluted four times with water, transferred to chromatographic glass vials, and analyzed.

### 2.6. Plasma and Erythrocytes Samples Analysis

The LC-MS/MS analysis was performed using the Acquity UPLC system (Waters, Milford, MA, USA) equipped with cooled autosampler. The sample temperature in an autosampler was 6 °C and the injection volume was 2 µL. The Waters BEH Shield C18 column (1.7 µm, 2.1 × 10 mm) was thermostatted in a column oven at 60 °C. The flow rate was 0.350 mL/min, and a total run time was 8 min. Eluents: A: water with 0.1% formic acid (FA), B: methanol with 0.1% FA. The following gradient was used: 0.0 min–3% B, 2.5 min–14% B, 4.6 min–60% B, 4.8 min–90% B, 6.1 min–3% B. 

MS analysis was performed using the SYNAPT G2 Si mass spectrometer (Waters, Milford, MA, USA) equipped with electrospray ionization source (ESI) in a positive ionization mode. The spray voltage, source temperature and the de-solvation temperature were set at 0.5 kV, 140 °C, and 450 °C, respectively. Data acquisition was performed using the MassLynx software (Waters) for the following ions (m/z): 150.0919, 156.1295, 237.1239, 243.1339, 263.1090, 267.1382, 279.1457, 286.1897, 307.1770, and 314.2209 for DMA, D6-DMA, ornithine, D6-ornithine, citrulline, D4-cytrulline, Arginine, D7-Arginine, ADMA, symmetric dimethylarginine (SDMA), and D7-ADMA, respectively. Standard calibration curves were prepared using the following concentration ranges: 3 to 150 μM for ornithine, 5 to 250 μM for Arginine, 0.05 to 2.5 μM for ADMA and SDMA, 1 to 50 μM for citrulline, and 0.14 to 7 μM for DMA.

### 2.7. Laser Doppler

All the individuals underwent the laser Doppler flowmetry (LDF) measurements using the Perimed PeriFlux System 5000, Sweden, with a PeriFlux heating unit, performed strictly according to the manufacturer’s instructions. The protocol was adapted from our previous study [12]. First, all the individuals were advised to rest for 15 min in horizontal position in a quiet laboratory at the room temperature 22 °C.

Afterward, their forearm was fixed by a vacuum pillow and the laser probe was placed on a skin, where no superficial veins were visible. The baseline flow and values were obtained within the first 10 min with a probe heated to 33 °C. It was subsequently heated to 43 °C by the PeriFlux heating unit for the next 30 min of protocol. According to the previous studies [13,14,15,16], there are at least two independent mechanisms that are involved in the skin microvascular vasodilatation in response to local heating. First—a peak observed in laser Doppler flowmetry is caused by the fast-responding vasodilator axon reflex activated by heat-sensitive receptors on afferent nerves mediated by antidromic release of a vasodilatory neurotransmitters (calcitonin gene-related peptide (CGRP), substance P) [17]. After initial brisk increase, the plateau phase is observed, which is followed (after 10–20 min) by slowly responding vasodilator system based on nitric oxide production during prolonged local heating. In order to evaluate the change in the cutaneous blood flow, the maximum heating index (MHI) was counted. It is the ratio between areas under the curve of 10 min blood flow during maximum heating (late NO-dependent phase) to 10 min baseline flow (before heating). Using the MHI better reflects nitric-oxide dependent endothelial function, reducing the overlap influence of autonomic system.

### 2.8. EndoPAT

The profile of endothelial function was assessed using the EndoPAT 2000 device (Itamar Medical, Caesarea, Israel). The EndoPAT detects plethysmographic pressure changes in the fingertips, caused by the arterial pulse, where special sensors are placed and translates it to a peripheral arterial tone (PAT). After 6 min of basal register, the occlusion by sphygmomanometer cuff inflated 40 mmHg over systolic pressure was made. Occlusion measurement was done with additional 5 min of post occlusion measurement (hyperaemic period). The PAT values obtained from contralateral limb serve as a control to exclude the individual systemic changes in blood flow. The achieved data were calculated using a computerized automated algorithm provided with the device. The results were compared using the reactive hyperaemia index (RHI) and its logarithm (lnRHI), which corresponded with the endothelium-mediated changes in vascular tone after occlusion and reflected the arterial endothelial function. Additionally, the augmentation index (AI) was assessed by analyzing the two pressure peaks signals from ascending aorta generated during the cardiac cycle. The first peak was derived from the pulse wave generated by the left ventricle, where the second was the reflection from arterial walls which superimpose to the first ventricular wave. AI is defined by the difference in these pressures, expressed as a percentage of the measured pulse pressure [18]. Its value depends on the vascular stiffness and aorta elasticity. Since it was shown that the augmentation index is heart rate dependent, a more reliable indicator is the augmentation index normalized for a heart rate of 75 bpm (AI-75) [19]. The whole protocol was conducted in pursuance of the manufacturer’s instructions. According to the Bonetti et al. study, the RHI cut-off value was set at RHI < 1,67 referring endothelial dysfunction [20]. All experiments were performed in an air-conditioned, quiet room with a constant air temperature of 20 °C.

### 2.9. Statistical Analysis 

The data are presented as the mean ± SD. The differences between two continuous parameters were assessed using a Mann–Whitney U-test or a Student’s *t*-test, following the Shapiro–Wilk test and Levene’s test as appropriate. Additionally, Spearman’s rank correlation coefficient was performed. All calculations were made with Statsoft^®^ Statistica 13.3 software, Krakow, Poland and Graph Pad PRISM^®^ 8.4 San Diego, CA, USA. 

## 3. Results

### 3.1. Baseline Characteristics

The study diabetic subjects and healthy controls were matched with respect to the age and sex distribution. There were, however, significant differences in the waist to hip ratio (WHR), weight, body mass index, lipid and glucose metabolism (Table 1). Additionally, higher levels of high-sensitive CRP and uric acid with decreased magnesium and sodium concentration were noted in the diabetic subjects. As compared to healthy individuals, patients suffering from DM had significantly higher HOMA-IR and lower QUICKI indexes. 

### 3.2. Assessment of Endothelial Function

**Figure 1:**
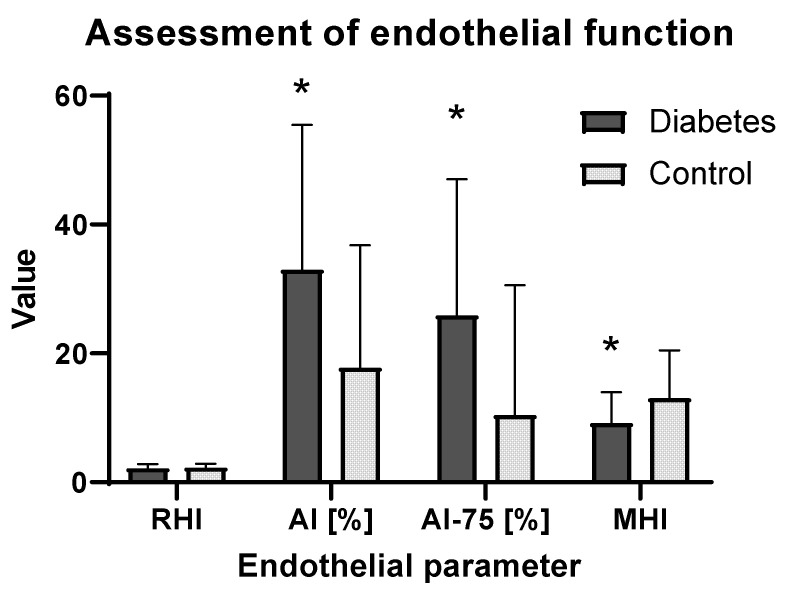
Assessment of endothelial function by EndoPAT 2000 and Laser Doppler Flowmetry. Abbreviations: RHI: reactive hyperaemia index (EndoPAT 2000); AI (%): augmentation index (EndoPAT 2000), AI-75 (%): augmentation index normalized for a heart rate of 75 beats/min (EndoPAT 2000), MHI: maximum hyperaemia index (Laser Doppler Flowmetry). Correlation of endothelial function with NO and biochemical metabolites in DM patients. *- *p* < 0.05 vs. control.

Assessment of endothelial function by EndoPAT 2000 indicated a significantly higher augmentation index (AI) and the AI normalized for a heart rate of 75 beats/min in diabetic patients. As compared to healthy individuals, diabetics had a decreased vascular response observed in the laser Doppler flowmetry, as reflected by decreased maximum hyperaemia index (MHI). The endothelial function assessment is shown in Table 2 and Figure 1.

### 3.3. Parameters of the Nitric Oxide Bioavailability in Erythrocytes and Plasma

Three of the six evaluated metabolites of the nitric oxide pathway were found to be significantly different in the plasma compartment (Arginine, DMA, citrulline). One was found to be altered in the erythrocytes (citrulline), when compared to the control group. The citrulline level was decreased in both compartments among DM patients, which was more noticeable in the erythrocytes. Simultaneously, the Arginine level was reduced in plasma, with no differences in the erythrocyte levels between groups. On the contrary, the dimethylamine (DMA) concentration was increased in plasma which was consecutively not observed in red blood cells (RBCs). All of the alterated nitric oxide metabolites were found at higher concentrations in plasma in both groups.

**Figure 2:**
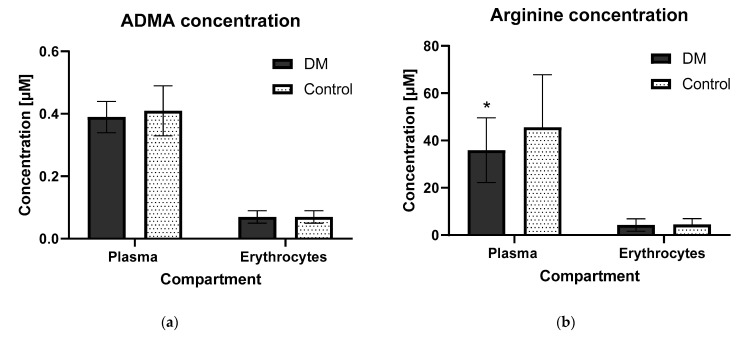

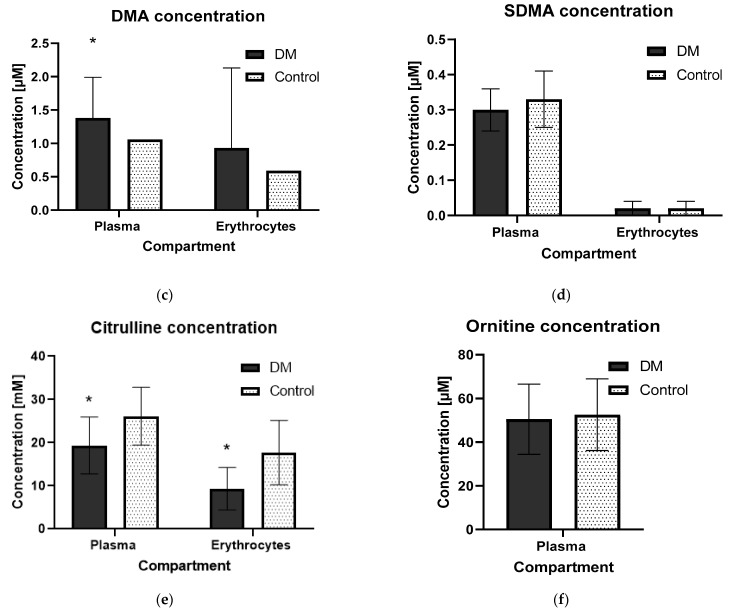
(**a**–**f**) Nitric oxide metabolites concentration in different compartments. *- *p* < 0.05 vs. control.

**Figure 3:**
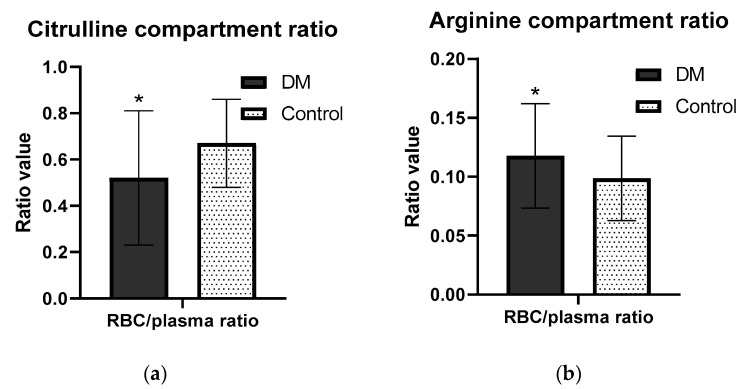
(**a**,**b**) Citrulline and Arginine compartment ratios. *- *p* < 0.05 vs. control.

**Figure 4:**
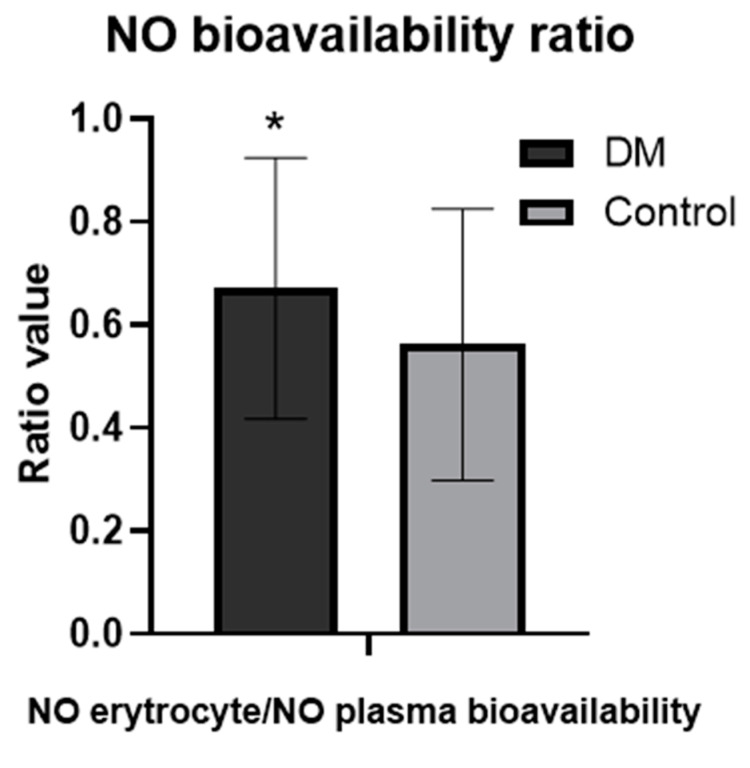
Nitric oxide (NO) bioavailability ratio. *- *p* < 0.05 vs. control.

In order to define the involvement of particular pathways in the alterations of the nitric oxide biotransformation the product to substrate ratios both, in the plasma and erythrocyte compartment in particular reactions were assessed. The study revealed significantly increased ornithine/Arginine and decreased ADMA/DMA ratio in the plasma of DM subjects. Additionally, reduced Arginine/ADMA ratio was noted in plasma with preserved proportion in erythrocytes. By comparing these ratios, higher NO bioavailability in the RBC of diabetic subjects was also identified. Futhermore, Arginine and citrulline compartmental concentration ratio were proved to be statistically different between the groups (Table 3 and Figure 2, Figure 3 and Figure 4).

### 3.4. Assessment of the Relationship between Biochemical Results and Endothelial Function

**Figure 5:**
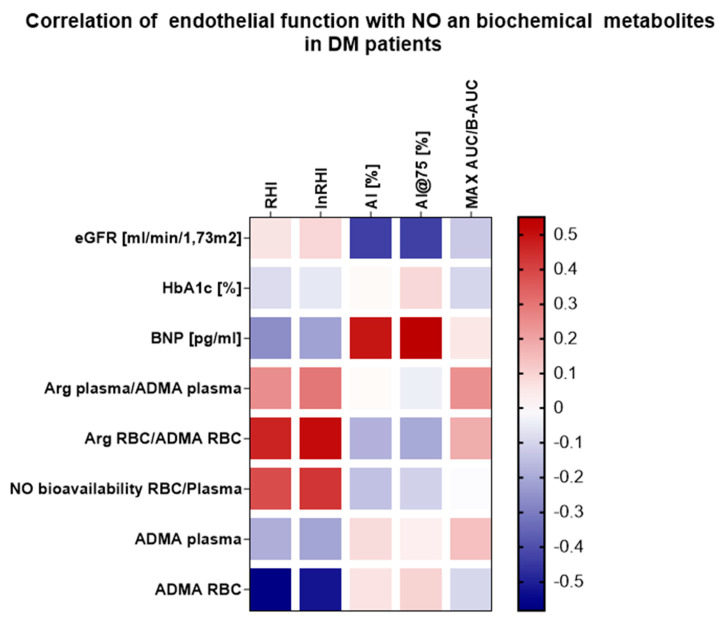
Correlation of endothelial function with NO and biochemical metabolites in DM patients. Abbreviations: RHI: reactive hyperaemia index (EndoPAT 2000); AI (%): augmentation index (EndoPAT 2000), AI-75 (%): augmentation index normalized for a heart rate of 75 beats/min (EndoPAT 2000), MHI: maximum hyperaemia index (Laser Doppler Flowmetry); RBC: red blood cells; eGFR: estimated glomerular filtration rate; BNP: brain natriuretic peptide; ADMA: Asymmetric dimethylarginine; Arg: L-Arginine.

The analysis of biochemical results and endothelial function revealed a moderate correlation between AI and eGFR and BNP. A similar correlation was found between endothelial function measured using EndoPAT and ADMA concentration, ADMA/Arginine ratio, and NO bioavailability in the RBC compartment with no such dependence in the plasma of DM patients (Figure 5).

## 4. Discussion

To our knowledge, this is the first research that evaluates the nitric oxide metabolic pathway both, in erythrocyte and plasma compartments. Furthermore, the erythrocyte nitric oxide metabolism and endothelial dysfunction in diabetic subjects were assessed. Recent findings emphasize the key role of endothelial dysfunction in diabetic patients as the first step leading to the development of vascular complications and organ damage. 

Insulin resistance, a hallmark of metabolic syndrome, impairs vascular response and increases cardiovascular risk. Involvement of insulin resistance and endothelial dysfunction in pathological disorders contribute to impairment in the NO-dependent vasodilatation, cellular glucose uptake, enhancement in oxidative stress, and inflammation, leading finally to atherosclerosis. Strong association of insulin and endothelial signaling disturbances contributes inflammation, disrupting the balance between vasodilating–vasoconstrictive endothelial mechanisms as well as between the insulin-dependent PI3-K/Akt–MAPK/ERK pathways [21].

**Figure 6:**
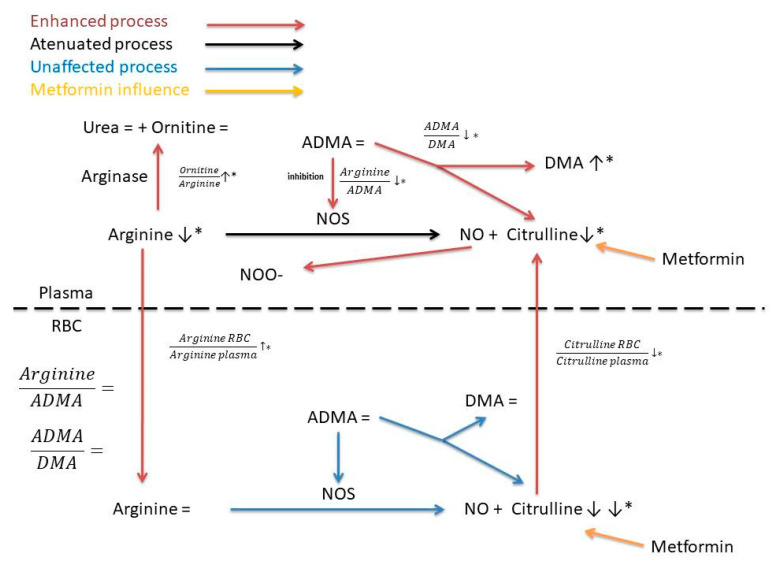
Nitric oxide metabolism alteration in different compartments. Abbreviations: ADMA: asymmetric dimethylarginine; DMA: dimethylamine; NOS: nitric oxide synthase; NO: nitric oxide RBC: erythrocyte SDMA: symmetric dimethylarginine. *- *p* < 0.05 vs. control.

For many years erythrocytes have been considered to be a natural sink for the NO molecules because of hemoglobin abundance which easily scavenge NO. However, recent studies give more evidence that erythrocytes’ role is far more complex, and they appear to be an important source of NO. In line with the studies that confirmed the activity of NOS in erythrocytes, the research proved the existence of nitric oxide metabolites and enzymatic inhibitors in RBCs. Besides citrulline concentration, no other alterations in the nitric oxide metabolites in erythrocytes of diabetic patients were found. It indicates that erythrocytes remain a buffer which is less affected by the deviations connected with endothelial dysfunction and that the synthesis of nitric oxide in erythrocytes remain unaltered in early diabetic individuals. Nitric oxide bioavailability in different compartments was also compared using the Arginine/asymmetric dimethylarginine ratio, which is a well-established marker of global Arginine bioavailability and the NO production. The analysis revealed its significantly higher values in the RBC which points out, even more distinctly, the importance of erythrocytes in preserving NO production in DM (Figure 6).

Contrary to the RBC compartment, substantial abnormalities in the NO synthesis in the plasma of DM patients were confirmed. First of all, a significantly reduced Arginine concentration was shown. It may be caused by shunting the L-Arginine from the eNOS to the arginase pathway with its enhanced arginase degradation, as an increased ornithine to Arginine ratio in plasma was found [18,22,23]. It is noteworthy that the results are in accordance with the studies by Shemyakin et al., proving that the downregulation of arginase improves endothelial function among patients with DM and may become a promising therapeutic target [24]. 

Additionally, enhanced Arginine transport from plasma to erythrocytes were found as Arginine compartment ratios were significantly different. Increased transport via system y+ in human erythrocytes had already been confirmed in different diseases affecting nitric oxide transformation, such as renal failure and chronic heart diseases [25,26]. It may indicate that the same upregulation of y+ transport occurred in the DM group as a compensatory response to maintain the NO production. 

Furthermore, low level of Arginine leads to the NOS uncoupling and results in excessive reactive oxygen species (ROS) formation, which also stems from NADPH oxidase (NOX) and mitochondrial complexes reactions [27]. As a result, the NO bioavailability is reduced in different ways—by decreased production because of substrate depletion and increased elimination through reaction with reactive oxygen species.

Moreover, significantly reduced Arginine to ADMA ratio in the DM group was also revealed, pointing at reduced NOS action and a subsequently diminished NO production. As no difference in ADMA concentration per se was found nor an increased DMA level, it collectively points at higher ADMA turnover in DM patients. Current literature is inconclusive in the assessment of dimethylarginine.

Dimethylaminohydrolase (DDAH) activity in diabetics, which is related with ADMA concentration. Numerous studies suggest that the high glucose level accompanied by increased concentration of proinflammatory cytokines, such as tumour necrosis factor (TNF), results in decreased DDAH activity and consequent increased ADMA level [28,29,30,31,32]. Our study cohort was composed of early-stage diabetic patients treated with metformin without the micro- and macrovascular complications. The low HbA1c levels reflect good glucose control, which may progressively result in preserved DDAH activity. This observation is consistent with the Xiong et al. study, proving the correlation of ADMA level with the macroangiopathy occurrence but not with the disease duration [33]. Additionally, DM patients were treated with metformin, which is a structural analog of ADMA reducing its concentration, possibly in the DDAH-dependent manner [34,35,36,37]. Furthermore, metformin reduces the advanced glycation end-products (AGEs) concentration what additionally restores the NO bioavailability [38]. 

Significantly lower plasma values of the citrulline in DM patients resulting from reduced eNOS activity caused by depletion of Arginine and increased inhibition were also found. Additionally, the use of metformin reduces citrulline levels [39,40]. According to Breier, M. et al. studies, short-term therapy with metformin reduces citrulline concentration by mean of 24% which is in compliance with results of this study—a 26% depletion on average of citrulline concentration between groups [39]. The mechanism of metformin-dependent citrulline reduction is not clear. Some authors seek the mechanism in urea cycle activity changes, inhibition of mitochondrial complex I, increased urinary excretion by kidneys, or decreased gut absorption associated with gastrointestinal side effects of metformin [39,41,42,43]. Reduced citrulline concentration in the erythrocyte compartment, more pronounced in the DM group was also noted.

In this study, the endothelial function using the two independent methods was also evaluated. First, the vascular response was assessed using the peripheral arterial tonometry with a post-occlusive reactive hyperaemia (PORH) measured as a reactive hyperaemia index (RHI). Second, with the laser Doppler flowmetry (LDF). A diminished endothelial function was found only by using the second method. 

The pathophysiology of the vascular relaxation in response to numerous stimuli (heating or occlusion) is different and may explain to some extent, why only local thermal hyperemia (LTH) indicates endothelial dysfunction in the diabetic subjects. The sensory nerves response and endothelium-derived hyperpolarizing factors have been described as the major points affecting both, the initial peak and the following increased blood flow after occlusion. Putative elements affecting PORH include the cytochrome epoxygenase metabolites and the large-conductance calcium-activated potassium channels (BKCa) in the vascular smooth muscle cells and in sensory nerves [44,45,46]. Hence, compared to other mechanisms regulating vascular response, the role of nitric oxide pathway in the post-occlusion response turned out not to be so crucial [47,48].

On the contrary, in the LDF response to heating stimuli, nitric oxide is responsible for vascular response in approximately 70% [49]. The first phase is mediated by a local sensory nerve axon reflex and the plateau depends mostly on the NO [45,49]. The maximum hyperemia index (MHI) was used to eliminate the neuronal influence. It reflects the nitric oxide-dependent plateau phase and expresses the endothelial function. Applying MHI results in a higher sensitivity and explains the discrepancies between the LTH and PORH outcomes. The results of this research are in line with Faisel and Heier studies, who confirmed endothelial dysfunction in diabetes mellitus type 1 group using LDF with intact vascular reaction in peripheral arterial tonometry [50,51].

The results showed that the augmentation index (AI) in diabetics was statistically higher, strictly corresponding with the increased vascular stiffness and the reduced flexibility of the aorta among diabetic patients Additionally, the AI-75 positively correlated with BNP concentration (*r* = 0.55) and negatively with the eGFR value (*r* = −0.43). It reflects that arterial stiffness in DM patients appears at the early stage of the disease and induces the organ-mediated complications from the beginning.

The nitric oxide metabolic pathway was also compared by correlating the products’ concentrations with the endothelial function results. The analysis confirmed that the endothelial function in DM patients correlated with NO bioavailability and NOS activity more in the RBC compartment than in plasma (*r* = 0.38 and *r* = 0.46). Furthermore, similar dependency was found within the erythrocyte ADMA concentration (*r* = −0.58) (Figure 2). Hence, it may be postulated that erythrocytes play an important role in compensating the endothelial dysfunction occurring in DM. Further studies are needed to evaluate their specific position in the NO metabolism and to define novel therapeutic targets to prevent the DM micro- and macrovascular complications.

## 5. Conclusions

Patients at an early stage of DM revealed endothelial dysfunction, which could be diagnosed earlier using the laser Doppler flowmetry. This group of subjects showed significant differences in the nitric oxide metabolism, which was more pronounced in the plasma compartment. The research proved decreased NOS activity with aggravated ADMA degradation and Arginine depletion. It indicated that erythrocytes remain a buffer less affected by the DM deviations with higher NO bioavailability than in the plasma compartment. Additionally, it was revealed that endothelial dysfunction correlates to a greater extent with abnormalities of nitric oxide pathway noted in RBC than in plasma compartment. The disclosed findings show the importance of the RBC as a NO-buffer. Hence, the RBCs play a key-role in maintenance a healthy vascular status in those with early diabetes mellitus. Additional studies in that field should be performed in order to extend the knowledge regarding the RBC NO-buffer, which may be used in the prevention or treatment of vascular complications in the diabetic group.

## 6. Limitations

Several limitations of this study should be addressed. The first limitation regards the measured molecules. As NO easily reacts with hemoglobin it was not possible to measure it directly and our results are mainly based on by-products which are more stable. Furthermore, different permeability of erythrocyte membrane may affect compartment distribution and study outcomes. The separation process might affect the analytes equilibrium, therefore additional experiments should be conducted to assess the potential significance of that process. In this study, subjects with newly onset of diabetes, without concomitant cardiovascular disorders, were investigated. Hence, the results of this study cannot be simply extrapolated to the whole spectrum of diabetic population.

## Figures and Tables

**Table 1:** Demographic and biochemical characteristics between studied groups including cardiovascular risk stratification parameters. Results are presented as mean ± SD.

Parameter	Diabetes Group *n* = 35 (Mean ± SD )	Control Group *n* = 45 (Mean ± SD )	*p* Value
Age (year)	59.80 ± 9.00	55.42 ± 10.75	NS
Women (%)	12 (34%)	19 (42%)	NS
Weight (kg)	88.37 ± 14.49	78.16 ± 14.43	<0.05
WHR	0.96 ± 0.08	0.91 ± 0.12	<0.05
BMI (kg/m^2^)	30.25 ± 4.07	25.80 ± 3.81	<0.05
WBC (k/μL)	7.12 ± 2.06	6.17 ± 1.46	NS
RBC (mln/μL)	4.71 ± 0.46	4.85 ± 0.58	NS
Hemoglobin (g/dL)	14.30 ± 1.24	14.58 ± 1.47	NS
Hematocrit (%)	42.04 ± 3.53	43.35 ± 4.68	NS
MCV (fL)	89.50 ± 4.33	89.48 ± 3.82	NS
MCH (pg)	30.47 ± 1.87	30.12 ± 1.41	NS
MCHC (g/dL)	33.99 ± 1.03	33.68 ± 1.13	NS
Platelets (k/μL)	253.27 ± 55.92	244.33 ± 55.69	NS
Glucose (mg/dL)	116 ± 39.11	93.30 ± 13.47	<0.05
HbA1 c (%)	6.14 ± 0.57	5.60 ± 0.30	<0.05
Insulin (uU/mL)	9.17 ± 5.38	6.65 ± 3.49	<0.05
Total cholesterol (mg/dL)	194.00 ± 49.90	214.38 ± 46.73	NS
LDL (mg/dL)	113.14 ± 42.24	130.57 ± 37.74	NS
HDL (mg/dL)	49.83 ± 11.70	56.98 ± 13.76	<0.05
Triglycerydes (mg/dL)	169.14 ± 108.98	134.26 ± 69.21	NS
Urea (mg/dL)	34.12 ± 6.59	33.03 ± 8.25	NS
Creatinine (mg/dL)	0.92 ± 0.15	0.96 ± 0.17	NS
eGFR (mL/min/1.73 m(2))	83.94 ± 14.49	79.26 ± 10.93	NS
Uric acid (mg/dL)	6.26 ± 1.68	5.44 ± 1.39	<0.05
Sodium (mmol/L)	139.41 ± 1.78	140.36 ± 2.42	<0.05
Potassium (mmol/L)	4.31 ± 0.28	4.22 ± 0.35	NS
Magnesium (mg/dL)	1.93 ± 0.23	2.12 ± 0.16	<0.05
Calcium (mmol/L)	9.70 ± 0.37	9.58 ± 0.38	NS
hsCRP (mg/L)	2.43 ± 2.42	1.94 ± 4.67	<0.05
TSH (ulU/mL)	1.66 ± 0.95	1.50 ± 0.67	NS
Alat (U/L)	27.53 ± 10.97	27.18 ± 12.63	NS
hsTroponin I (pg/mL)	2.31 ± 2.56	1.32 ± 1.53	NS
BNP (pg/mL)	29.16 ± 38.05	17.68 ± 15.28	NS
HOMA-IR	2.65 ± 1.56	1.58 ± 0.82	<0.05
HOMA beta	87.64 ± 119.41	90.17 ± 49.21	NS
QUICKI	0.34 ± 0.03	0.36 ± 0.03	<0.05

Abbreviations: NS: result statistically non-significant; BMI: body mass index; WHR: waist–hip ratio; WBC: white blood cells; RBC: red blood cells; MCV: mean (red blood) cell volume; MCH: mean corpuscular hemoglobin; MCHC: mean corpuscular hemoglobin concentration; PDW: platelet distribution width; eGFR: estimated glomerular filtration rate; HDL: high-density lipoprotein; LDL: low-density lipoprotein; hsCRP: high-sensitivity C-reactive protein; TSH: thyroid-stimulating hormone; BNP: brain natriuretic peptide.

**Table 2:** Assessment of endothelial function by EndoPAT 2000 and the Laser Doppler Flowmetry.

Parameter	Diabetes (Mean ± SD)	Control (Mean ± SD)	*p* Value
RHI	2.19 ± 0.62	2.28 ± 0.59	NS
AI (%)	32.97 ± 22.50	17.76 ± 19.00	<0.05
AI-75 (%)	25.86 ± 21.18	10.45 ± 20.15	<0.05
MHI	9.17 ± 4.82	13.04 ± 7.44	<0.05

Abbreviations: NS: result statistically non-significant; RHI: reactive hyperaemia index (EndoPAT 2000); AI: augmentation index (EndoPAT 2000), AI-75 (%): augmentation index normalized for a heart rate of 75 beats/min (EndoPAT 2000), MHI: maximum hyperaemia index (Laser Doppler Flowmetry).

**Table 3:** The Nitric oxide pathway metabolites ratios.

Plasma Ratios	DM Group (Mean ± SD)	Control Group (Mean ± SD)	*p* Value	RBC Ratios	DM Group (Mean ± SD)	Control Group (Mean ± SD)	*p* Value	Plasma/RBC Ratios	DM Group (Mean ± SD)	Control Group (Mean ± SD)	*p* Value
Arginine/ADMA	94.6 ± 40.3	116.5 ± 50.4	<0.05	Arginine/ADMA	65.03 ± 40.97	62.72 ± 34.66	NS	Citrulline _RBC_/Citrulline _plasma_	0.52 ± 0.29	0.67 ± 0.19	<0.05
ADMA/DMA	0.37 ± 0.23	0.47 ± 0.24	<0.05	ADMA/DMA	0.06 ± 0.04	0.07 ± 0.07	NS	Arginine _RBC_/Arginine _plasma_	0.12 ± 0.04	0.10 ± 0.04	<0.05
Ornitine/Arginine	1.69 ± 0.90	1.18 ± 0.59	<0.05								

Abbreviations: RBC: red blood cells.

## Data Availability

The original data used to support the findings of this study are available from the corresponding author upon request.

## Abbreviations

ADMA	asymmetric dimethylarginine
AGEs	advanced glycation end-products
AI	augmentation index
AI-75 [%]	augmentation index normalized for a heart rate of 75 beats/min
Arg	L-Arginine
BCl	benzoyl chloride
BMI	body mass index
BNP	brain natriuretic peptide
CVD	cardiovascular disorders
DDAH	Dimethylaminohydrolase
DM	Diabetes mellitus
DMA	dimethylamine
eGFR	estimated glomerular filtration rate
ER	estrogen receptors
ESC	European Society of Cardiology
HbA1c	glycated hemoglobin
HDL	high-density lipoprotein
hsCRP	high-sensitivity C-reactive protein
LDF	laser Doppler flowmetry
LDL	low-density lipoprotein
lnRHI	reactive hyperaemia index logarithm
LTH	local thermal hyperemia
MCH	mean corpuscular hemoglobin
MCHC	mean corpuscular hemoglobin concentration
MCV	mean (red blood) cell volume
MHI	maximum heating index
NO	nitric oxide
NOS	nitric oxide synthase
NOX	NADPH oxidase
NS	result statistically non-significant
PAT	peripheral arterial tone
PDW	platelet distribution width
PORH	post-occlusive reactive hyperaemia
RBC	red blood cells
RHI	reactive hyperemia index
ROS	reactive oxygen species
SDMA	symmetric dimethylarginine
TNF	tumour necrosis factor
TSH	thyroid-stimulating hormone
WBC	white blood cells
WHR	waist–hip ratio
